# Highly efficient up-conversion luminescence in Er^3+^/Yb^3+^ co-doped Na_5_Lu_9_F_32_ single crystals by vertical Bridgman method

**DOI:** 10.1038/s41598-017-09222-0

**Published:** 2017-08-18

**Authors:** Shinan He, Haiping Xia, Jianli Zhang, Yongsheng Zhu, Baojiu Chen

**Affiliations:** 1 Key laboratory of Photo-electronic Materials, Ningbo University, Ningbo, Zhejiang 315211 China; 2 College of Physics and Electronic Engineering, College of Chemistry and Charmaceutical Engineering, Nanyang Normal University, Nanyang, 473061 China; 3 Department of Physics, Dalian Maritime University, Dalian, Liaoning Province 116026 China

## Abstract

Er^3+^/Yb^3+^ co-doped Na_5_Lu_9_F_32_ single crystals with different concentrations of Yb^3+^ ions were prepared to investigate their phase structure, up-conversion (UC) properties and mechanism of UC luminescence by Bridgman method. Under 980 nm near-infrared (NIR) excitation, three sharp UC emission bands topping at green ~525 nm, ~548 nm and red ~669 nm were obtained in Er^3+^/Yb^3+^ doped Na_5_Lu_9_F_32_ single crystals which are attributing to the transitions of ^2^H_11/2_ → ^4^I_15/2_, ^4^S_3/2_ → ^4^I_15/2_ and ^4^F_9/2_ → ^4^I_15/2_, respectively. The quadratic dependence of pump power on UC emission indicated that two-photon process is in charge of the transition from excited state of Yb^3+^ ions to lower state of Er^3+^ ion in Na_5_Lu_9_F_32_ single crystals. The long-accepted mechanism for the production of red and green emissions through up-conversion (UC) under 980 nm excitation in Er^3+^/Yb^3+^ co-doped materials apply in the Na_5_Lu_9_F_32_ host was displayed. The enhancement of the red emission was observed due to a cross-relaxation (CR) process of the form: ^4^F_7/2_ + ^4^I_11/2_ → ^4^F_9/2_ + ^4^F_9/2._ Furthermore, an ideal yellowish green light performance could be achieved with 1.0 mol% Er^3+^ doped certain Yb^3+^ concentrations samples, and its external quantum efficiency approached to 6.80% under 5.5 Wcm^−2^ 980 nm excitation which can be applied in developing UC displays for electro-optical devices.

## Introduction

Considerable interests in rare-earth (RE) doped UC phosphors have burgeoned since the development of synthetic methods for creating highly processable UC materials with applications in biolabeling^1, 2^, sub-band gap energy harvesting in photovoltaics^3, 4^, and security printing^5^. Due to its potential applications in biological fluorescence and volumetric displays, much effort has been devoted to the investigation on UC in RE^3+^ doped materials over the past years. Recently, a few basic works have been reported in order to explain the origin of the relatively high UC efficiencies^6^, the ability to adjust the color purity through manipulating dopant concentrations^7^, and the special phenomenon that result from surface effects in nanoscale UC phosphors^8, 9^.

Among all the rare-earth ions, Er^3+^ ion is one of the best candidates as an activator in up-conversion luminescence on account for its longer lifetimes of metastable energy levels and more homogeneous energy level array. By employing Yb^3+^ ions as sensitizer into Er^3+^ doped materials, the UC luminescence performance can be improved because of a larger absorption cross section at NIR of Yb^3+^ and the high-efficiency energy transfer from Yb^3+^ to Er^3+^ ions^10–12^.

A number of researches on Er^3+^/Yb^3+^ co-doped systems have been focused on exploring the UC mechanisms and developing novel hosts in recent years^13^. In order to reduce the multi-phonon nonradiative relaxation and realize the high efficient UC luminescence, low maximum phonon energy hosts are required and necessary. Compared with the popular oxide materials, it is well-known that fluorides are more efficient hosts for RE^3+^ ions due to their low energy phonons to produce strong UC fluorescence. Up till now, fluorides like Na_5_Lu_9_F_32_ have been investigated widely^14–16^. However, the studied materials of fluoride Na_5_Lu_9_F_32_ mainly concentrate on nano-crystals and powders^17^, and the Er^3+^/Yb^3+^ co-doped UC materials have never been in-depth studied in Na_5_Lu_9_F_32_ single crystals. Compared with nano-crystal, single crystal provides higher transmission for lights and thermal stability as well as good chemical durability. There are scarce reports on Na_5_Lu_9_F_32_ single crystals because of the difficulty in the crystal growth. Thus, Na_5_Lu_9_F_32_ in form of single crystal is an excellent candidate matrix for Er^3+^/Yb^3+^ ions to investigate the UC luminescence spectra.

Generally, there exists the problem of the inhomogeneous distribution of RE ions in single crystal resulted from crystal growth. In this study, Na_5_Lu_9_F_32_ single crystal was chosen as a matrix of Er^3+^ and Yb^3+^ ions in view of the same valence state and the very near ionic radii between Lu^3+^ (0. 861 Å) and Er^3+^ (0.881 Å), Lu^3+^ and Yb^3+^ (0.858 Å), which resulted in relatively homogeneous concentration of Er^3+^ and Yb^3+^ in Na_5_Lu_9_F_32_ single crystal. The Na_5_Lu_9_F_32_ single crystal with homogeneous rare earths is extremely important for the practical application in optical device.

## Results and Discussions

### Characteristics of Na_5_Lu_9_F_32_ single crystal

**Figure 1:**
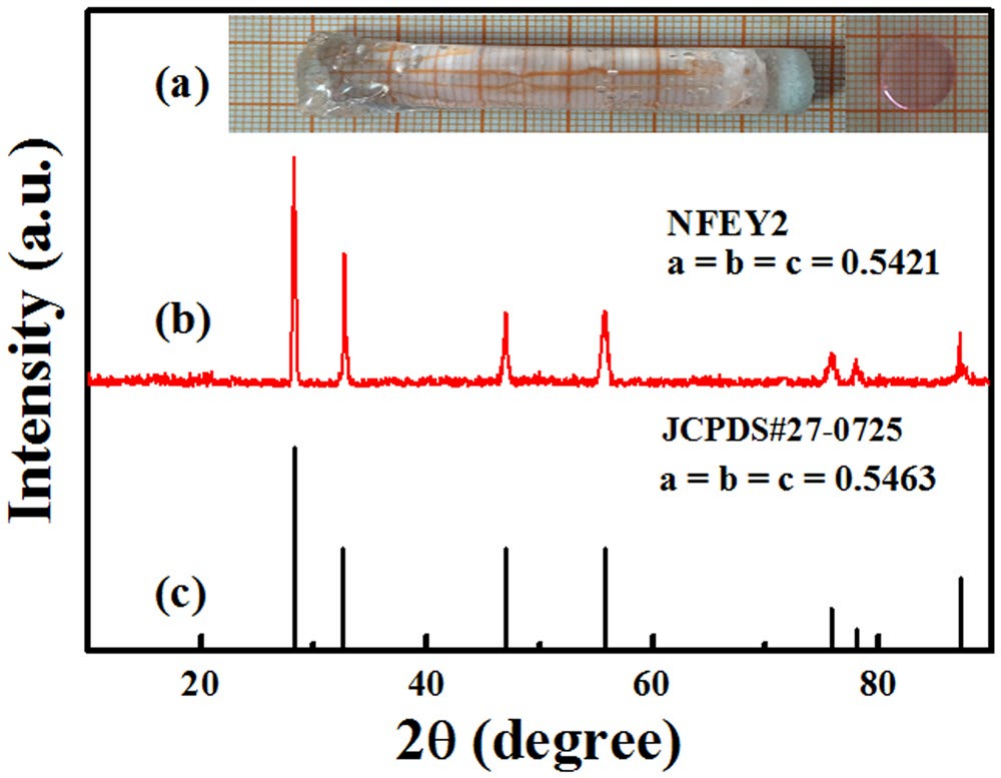
(**a**) The photo of Na_5_Lu_9_F_32_ single crystal, the left is boule of the crystal and the right is polished slice. (**b**) XRD patterns of the Na_5_Lu_9_F_32_: Er^3+^/Yb^3+^. (**c**) Standard line patterns of Na_5_Lu_9_F_32_ (27–0725).

In order to understand the structures and characteristics of the as-grown crystals, the crystallinity was confirmed in the X-ray powder diffraction (XRD) patterns as shown in Fig. 1(b). It can be seen from the XRD spectra of Er^3+^/Yb^3+^ co-doped Na_5_Lu_9_F_32_ single crystal in Fig. 1(b), the structure of the single crystal was confirmed to be cubic phase, in accordance with JCPDS card (27–0725) of Na_5_Lu_9_F_32_ as shown in Fig. 1(c), which the diffraction peak positions of the obtained samples doped with Er^3+^/Yb^3+^ ions are matched perfectly with those of standard Na_5_Lu_9_F_32_, though there exists a slight shift in the range of 0.10° to 0.45° as the dopant change, indicating that this transparent crystal has pure cubic phase. Moreover, the cell parameters were calculated by the following formula^18^

1 $$d=\frac{a}{\sqrt{{h}^{2}+{k}^{2}+{l}^{2}}}$$

Accordingly, the cell parameter is calculated to be a = 0.5421 nm (space group Fm3m) for sample NFEY2 (0.99 mol% Er^3+^/1.97 mol% Yb^3+^ sample) from the measured XRD patterns.

### Absorption and transmittance spectra of the Er^3+^/Yb^3+^ co-doped Na_5_Lu_9_F_32_ single crystal

**Figure 2:**
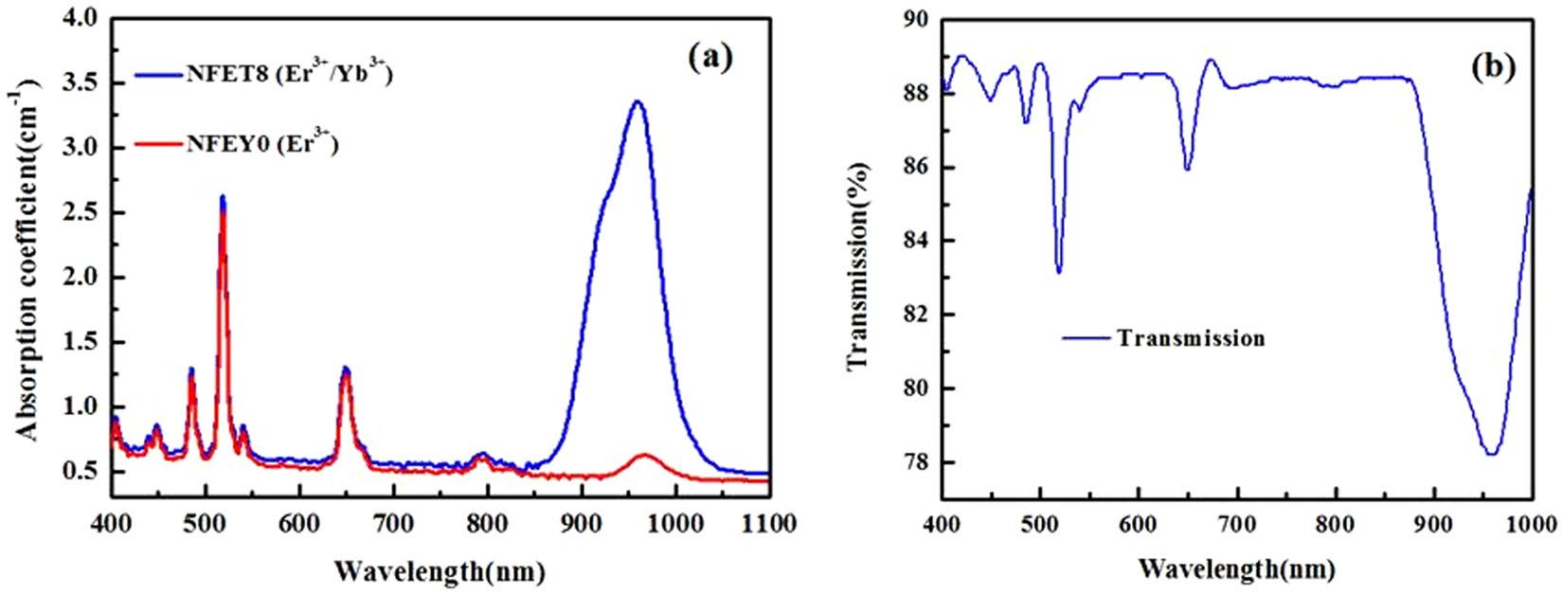
(**a**) Absorption spectra of Er^3+^/Yb^3+^single doped and co-doped Na_5_Lu_9_F_32_ single crystals. (**b**) Transmittance spectra of Er^3+^/Yb^3+^ co-doped Na_5_Lu_9_F_32_ single crystal.

The absorption spectra in the 400–1100 nm wavelength of the as-grown Na_5_Lu_9_F_32_ single crystal samples has been measured and presented in Fig. 2(a). The absorption peaks corresponding to transitions from the ^4^I_15/2_ ground state to excited states of the Er^3+^ ions are also assigned in Fig. 2(a). Compared the Er^3+^ singly-doped sample with Er^3+^/Yb^3+^ co-doped sample, there is nearly no intensity and position change for the shape of peaks in the visible region, where confirms that Er^3+^ ions can absorb visible light. As for the absorption center at 980 nm, Er^3+^/Yb^3+^ co-doped sample shows an obvious peak located at 980 nm corresponding to the transition from ^2^F_7/2_ → ^2^F_5/2_ of Yb^3+^ ion, which demonstrates that Yb^3+^ ions acts as an excellent sensitizer to absorb the pump light. The corresponding UV-VIS-NIR transmittance spectrum of well polished Er^3+^/Yb^3+^ co-doped Na_5_Lu_9_F_32_ single crystal with 1.5mm thickness also showed in Fig. 2(b). It can be confirmed that the transmittance was measured to be ~89% in the visible region (Na_5_Lu_9_F_32_ single crystals are highly transparent as shown in Fig. 1(a)), which proves that there exists a high transmittance in the range of 400–1000 nm.

### Emission spectra and energy transfer of as-prepared Na_5_Lu_9_F_32_ single crystal co-doped 1.0% Er^3+^ and χ% Yb^3+^ (χ = 0, 2, 6, 8)

**Figure 3:**
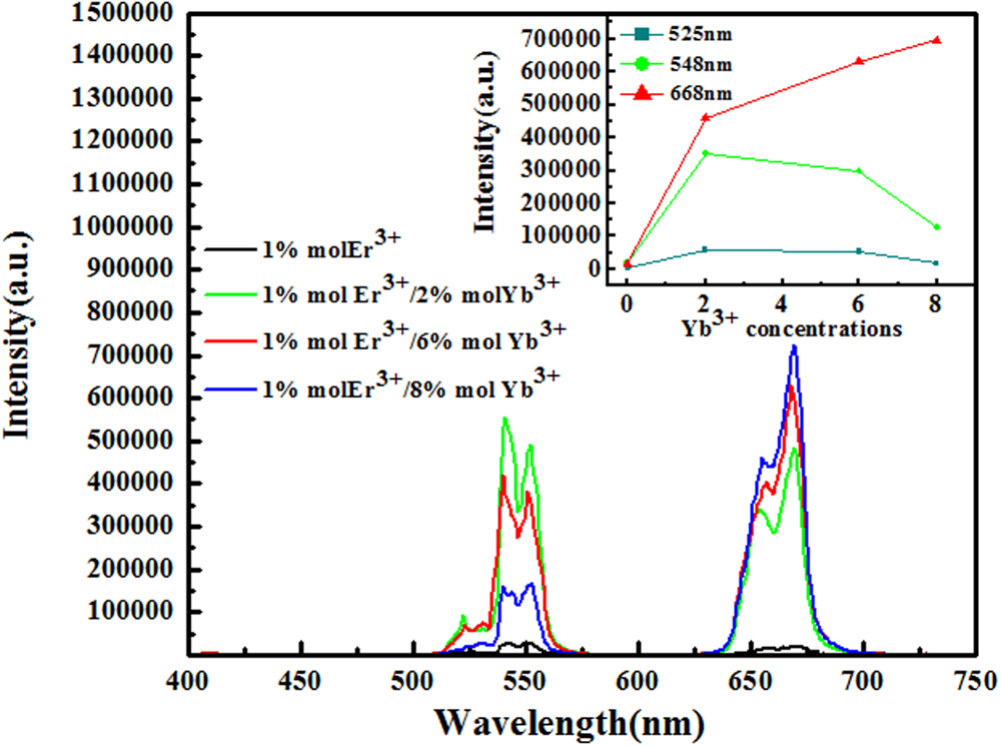
Comparison of the up-conversion luminescence spectra of Er^3+^/Yb^3+^ doped Na_5_Lu_9_F_32_ single crystals under 980 nm excitation at room temperature; the inset shows the integrated total intensity on Yb^3+^ concentrations in Er^3+^/Yb^3+^ doped Na_5_Lu_9_F_32_ single crystals.

The quantitative comparison of up-conversion luminescence of as-prepared Na_5_Lu_9_F_32_ single crystal co-doped 1.0% Er^3+^ and χ% Yb^3+^ (χ = 0, 2, 6, 8) under NIR 980 nm excitation have been shown in Fig. 3, from which three obvious bands in the range of 450–750 nm can be observed. As presented in Fig. 3, two green emissions around 525 nm and 548 nm are assigned to the radiative transition of ^2^H_11/2_ → ^4^I_15/2_ and ^4^S_3/2_ → ^4^I_15/2_, respectively. And red emission band from 638 to 688 nm is attributed to the ^4^F_9/2_ → ^4^I_15/2_ transition of Er^3+^ ion. Compared luminescence intensity of Er^3+^ single-doped sample with Er^3+^/Yb^3+^ co-doped Na_5_Lu_9_F_32_ one, there is a distinct improvement of up-conversion luminescence efficiency when the Yb^3+^ ions take part in. The relative amount of Yb^3+^ sensitizer ions tend to obviously improve the up-conversion luminescence (UCL) intensity in Er^3+^/Yb^3+^ phosphors, thus we have investigated the impact of various ytterbium concentrations on the intensity of the UC emission under the same experiment condition. The insert of Fig. 3 shows the integrated total intensity on Yb^3+^ concentrations. The UCL intensity of 668 nm emission increases rapidly as the Yb^3+^ concentration increases and reaches its maximum value when Yb^3+^ concentration is 7.97 mol% in present research, which is attributed to the aggravated cross-relaxation process of ^4^F_7/2_ + ^4^I_11/2_ − ^4^F_9/2_ + ^4^F_9/2_ with the increase of Yb^3+^ ions concentration. The concentration quenching of Yb^3+^ ions is not observed, while the maximum intensity of 548 nm and 525 nm emission is obtained when Yb^3+^ concentration is 1.99 mol%.

**Figure 4:**
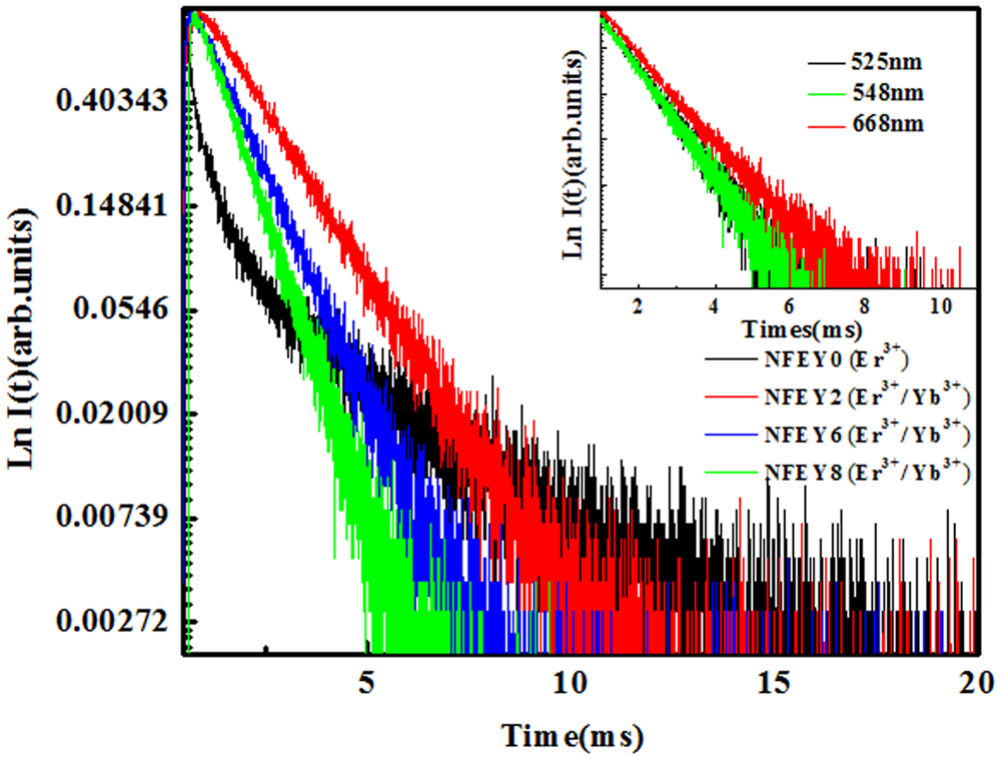
Decay curves of the Er^3+^/Yb^3+^ co-doped Na_5_Lu_9_F_32_ single crystals surveyed at 548 nm under 980 nm excitation, and the inset is the decay times centered at 525, 548 and 668 nm for 1.0% Er^3+^/8% Yb^3+^ co-doped Na_5_Lu_9_F_32_ single crystal.

To reveal the origin of these radiations and the mechanism of the UC emissions, the decay profiles of Na_5_Lu_9_F_32_ single crystal doped Er^3+^ with different amount of Yb^3+^ ions under 980 nm excitation at 548 nm is shown in Fig. 4, and the inset is the comparison of decay times centered at 525, 548 and 668 nm for 1.0% Er^3+^/8% Yb^3+^ co-doped Na_5_Lu_9_F_32_ single crystal. These decay profiles were fitted with non-exponential, the decay time of these radiations centered at 525, 548 and 668 nm show the similar value of ~880 μs from the inset of the Fig. 4. Meanwhile, the lifetime for 1.0% Er^3+^ singly doped Na_5_Lu_9_F_32_ single crystal and 1.0% Er^3+^/χ% Yb^3+^ (χ = 2,6,8) co-doped Na_5_Lu_9_F_32_ single crystals are τ_m_ = 311.1 μs, τ_m_ = 1567.7 μs, τ_m_ = 1114.4 μs, τ_m_ = 870.4 μs respectively. It suggests that there is no concentration quenching in present doping concentration. And it can be seen that the fluorescence for the co-doped crystals (NFEY2, 6, 8) decays more quickly than that for the single-doped one (NFEY0) from Fig. 4, which indicates an obvious enhancement of up-conversion luminescence efficiency arise from dopants of Yb^3+^ ions. As presented in Fig. 4, the lifetime of green emission decreased apparently when the concentration of Yb^3+^ ions increases, which can verify that cross-relaxation (CR) process of ^4^F_7/2_ + ^4^I_11/2_ → ^4^F_9/2_ + ^4^F_9/2_ occurred. The Na_5_Lu_9_F_32_ single crystal shows high incorporating concentrations for Er^3+^ and Yb^3+^. Owing to the similar radii size and same valence state of between rare earth ions, Er^3+^ ions and Yb^3+^ ions could easily take place of the position of Lu^3+^ in Na_5_Lu_9_F_32_ single crystal. The homogeneity of REs in Na_5_Lu_9_F_32_ can be reflected from the effective segregation coefficients of REs in single crystal and they can be estimated from the measured concentration and the formula $c=k{c}_{0}{(1-f)}^{(k-1)}$, where c and c_0_ express the concentrations of RE in single crystal and raw material, k is effective segregation coefficient of RE, and f is the crystallization rate^19^. The effective segregation coefficients for Er^3+^ and Yb^3+^ in Na_5_Lu_9_F_32_ were about 0.990 and 1.013, respectively, which approach to 1. It indicates that a relatively homogeneous concentrations of REs can be obtained because of the very near ionic radii between Lu^3+^ (0. 861 Å) and Er^3+^ (0.881 Å), Lu^3+^ and Yb^3+^ (0.858 Å) and results into high luminescence effect of Er^3+^ and Yb^3+^.

**Figure 5:**
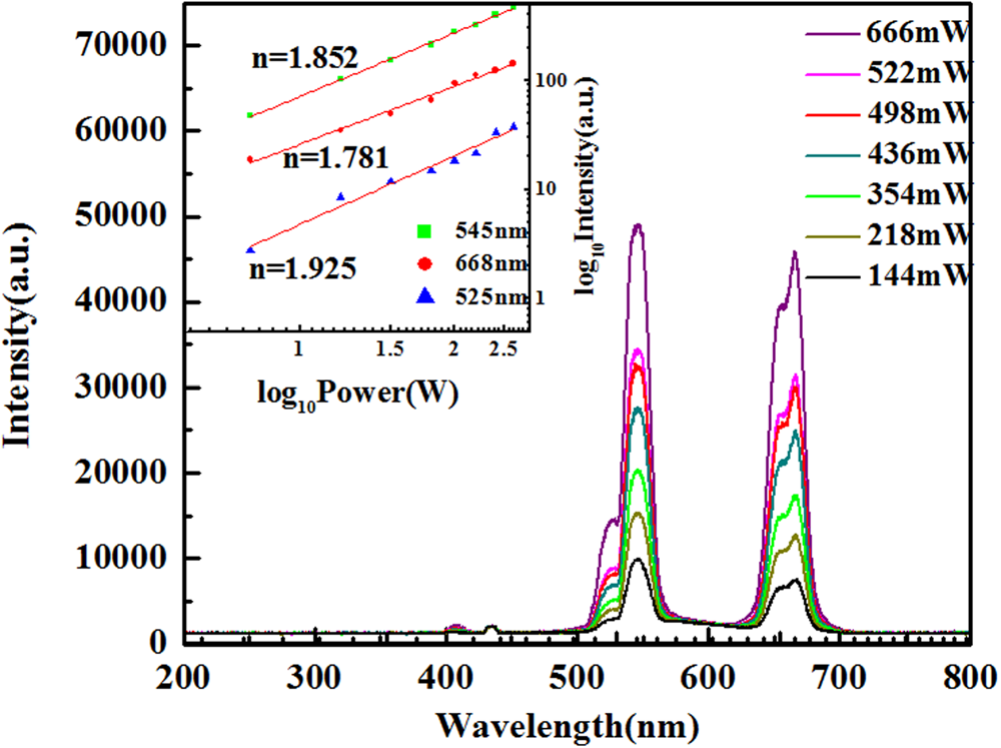
Log–log dependence of the up-conversion intensities of 1.0 mol% Er^3+^ and 7.97 mol% Yb^3+^ co-doped Na_5_Lu_9_F_32_ single crystal at 525,548, 668 nm emissions as a function of the excitation power at 980 nm.

In order to investigate the UC dynamics of Er^3+^/Yb^3+^ co-doped Na_5_Lu_9_F_32_ single crystal, the pump-power dependence of luminescence intensities was measured as a function of excitation power density under the 980 nm excitation. As shown in Fig. 5, the up-conversion luminescence intensities increased as excitation power increasing to the maximum ~666 mW, which may conclude that high excitation power can improve the up-conversion luminescence efficiency. It is known that the relation between emission intensity I_em_ and NIR excitation power I_em_∝P^n^ in frequency up-conversion process^20^, where n is the number of pump photons required to excite to the emitting state. From the log–log dependence of the integrated green (~525 and ~548 nm) and red emission (~668 nm) intensities on the excitation power at 980 nm shown in the inset of Fig. 5, the graph of log (*I*_em_) versus log (P) yields a slope of n equal to approximately 1.968, 1.852, 1.721, respectively. The value of n is approximately equal to 2, which implied the lower thermal effect and saturation effect in Na_5_Lu_9_F_32_: Yb^3+^, Er^3+^ single crystals. Thus, the quadratic dependence indicates that blue, green and red emissions all arise from two-photon UC processes.

**Figure 6:**
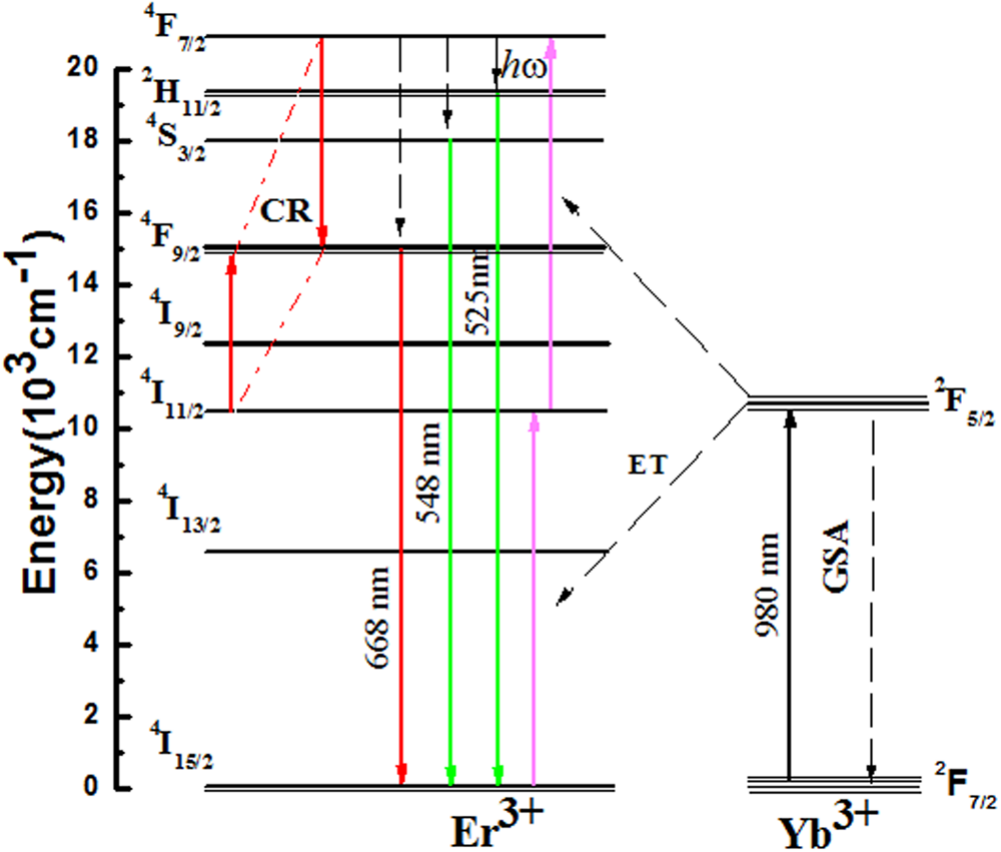
Energy level diagram of Er^3+^/Yb^3+^ and energy transfer progress.

To better understand the mechanism of UC luminescence in Er^3+^/Yb^3+^ co-doped Na_5_Lu_9_F_32_ single crystal, a schematic energy level diagram of Er^3+^/Yb^3+^ and the energy transfer process has been presented in Fig. 6. Following the 980 nm pumping light, the ^4^I_11/2_ level of Er^3+^ ion can be directly excited from ground state ^4^I_15/2_ or by energy transfer (ET) process from ^2^F_5/2_ level of Yb^3+^ to Er^3+^: ^2^F_5/2_ (Yb^3+^) + ^4^I_15/2_ (Er^3+^) → ^2^F_7/2_ (Yb^3+^) + ^4^I_11/2_ (Er^3+^). It should be noted that interactions between two Er^3+^ ions cannot necessarily be ignored. An NIR photon can populate an Er^3+^ ion to its ^4^I_11/2_ state directly. Another Er^3+^ ion also in the ^4^I_11/2_ state and in close proximity will transfer its energy to the initial ion, thereby exciting it to the ^4^F_7/2_ state. However, the absorption cross section of Yb^3+^ at ~980 nm is much larger than that of Er^3+^: ^4^I_11/2_, which can be seen from Fig. 2, resulting in the ET process from Yb^3+^ to Er^3+^ is dominant in the excitation of (Er^3+^) level. It is known that when co-doped samples are excited by high power successive laser at 980 nm, the Er^3+^ ion may decay non-radiatively from the ^4^I_11/2_ state to the ^4^I_13/2_ state following the initial energy transfer from the Yb^3+^ ion by multi-phonon relaxation in recent works. However, bridging the energy gaps of ^4^I_11/2_ → ^4^I_13/2_ (3619 cm^−1^) or ^4^S_3/2_ → ^4^F_9/2_ (3217 cm^−1^) requires at least 6−7 phonons, multi-phonon relaxation will rarely happen in Na_5_Lu_9_F_32_ single crystal due to its relatively low phonon energies about ~441 cm^−1^ ^21^. Thus, the excitation in the ^2^F_7/2_ → ^2^F_5/2_ transition of Yb^3+^ is followed by a two-step ET process to neighbouring Er^3+^ ions as shown in Fig. 6, which brings Er^3+^to the ^4^F_7/2_ level. The populated ^4^F_7/2_ level of Er^3+^ then non-radiatively relaxes fast to the lower ^2^H_11/2_ and ^4^S_3/2_ states because of the smaller energy gaps of ^4^F_7/2_ → ^2^H_11/2_ (1162 cm^−1^) and ^2^H_11/2_ → ^4^S_3/2_ (794 cm^−1^), and two green emissions then be observed. Above process generates two ^2^H_11/2_ → ^4^I_15/2_ and ^4^S_3/2_ → ^4^I_15/2_ transitions centered at 525 and 548 nm, respectively. Meanwhile, red emission band is attributed to the ^4^F_9/2_ → ^4^I_15/2_ de-excitation process. And the population on ^4^F_9/2_ should come from the CR process of ^4^F_7/2_ + ^4^I_11/2_ → ^4^F_9/2_ + ^4^F_9/2_, which can be evidenced by the Yb^3+^ concentration-dependent UCL spectra of Na_5_Lu_9_F_32_: Yb^3+^, Er^3+^ single crystals (Fig. 3) as well as the decay curves of the Er^3+^/Yb^3+^ co-doped Na_5_Lu_9_F_32_ single crystals surveyed at 548 nm (Fig. 4). As shown in the Fig. 3, When the concentration of Yb^3+^ increases to about 7.97 mol%, probably due to concentration quenching between Er^3+^ and Yb^3+^ ions, the energy transfer process of Er^3+^ (^4^I_11/2_) + Yb^3+^ (^2^F_5/2_) → Er^3+^ (^4^F_7/2_) + Yb^3+^ (^2^F_7/2_) reduced accordingly, which leads to the reduction of the population of ^4^F_7/2_ state of Er^3+^ ions and results in the decrease of 525 nm and 548 nm emissions. Thus the strongest emissions at 525 nm and 548 nm in this study is observed when the Yb^3+^ is about 1.99 mol%. However, the process of two photon UC process 2^2^F_5/2_ (Yb^3+^) + ^4^I_15/2_ (Er^3+^) → 2^2^F_7/2_ (Yb^3+^) + ^4^F_7/2_ (Er^3+^) continues to increase luminescence intensify as the Yb^3+^ concentration increases. The concentration quenching between Yb^3+^ and Yb^3+^ ions has not been observed in this study.

### UC external quantum efficiency of Na_5_Lu_9_F_32_: 1% Er^3+^, 7.9% Yb^3+^

**Figure 7:**
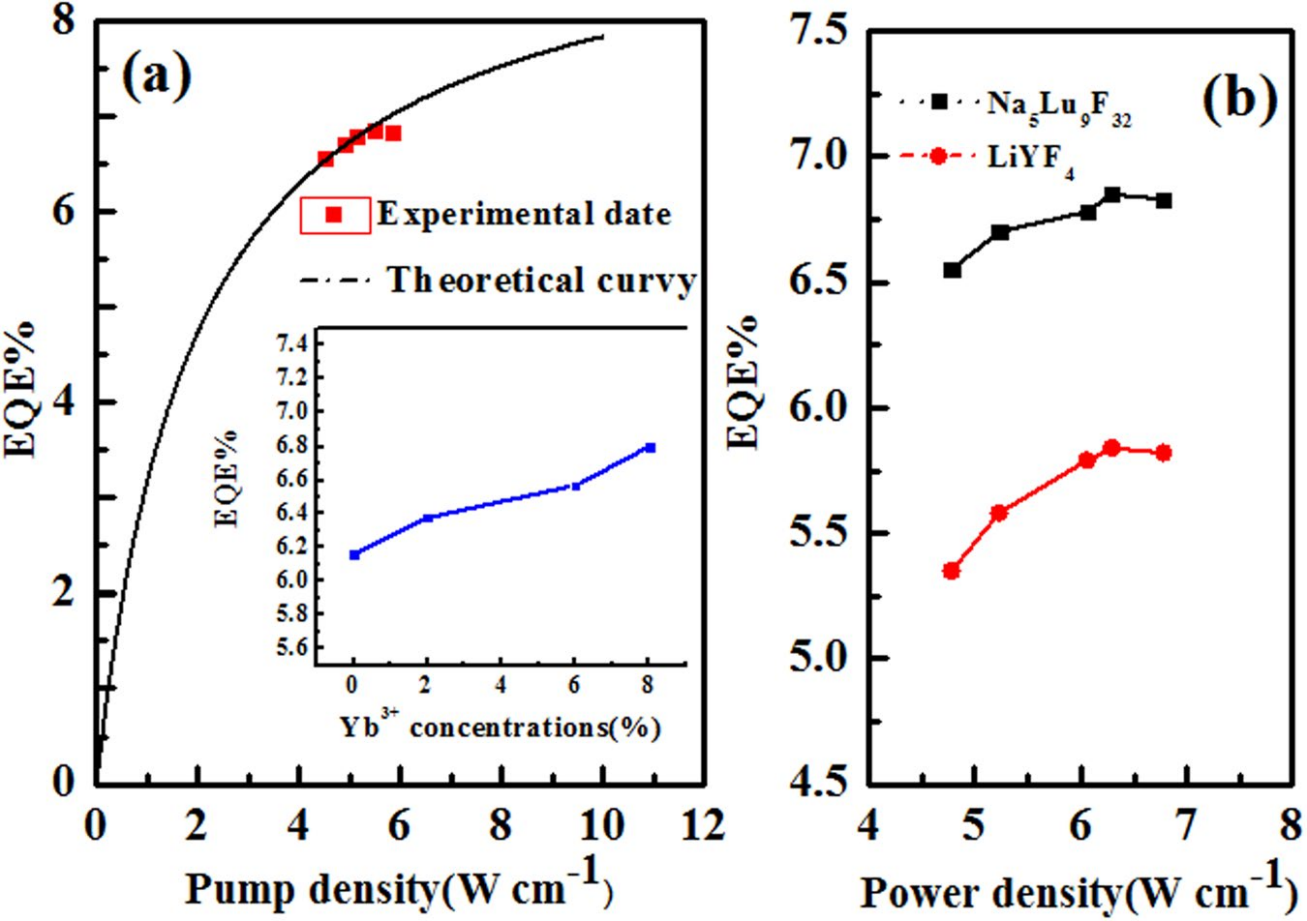
(**a**) Power-dependent UC external quantum efficiency at 980 nm light excitation in Er^3+^/Yb^3+^ doped Na_5_Lu_9_F_32_ single crystals, the inset shows the external quantum efficiency on Yb^3+^ concentrations; (**b**) Comparison of UC external quantum efficiency of Er^3+^/Yb^3+^ co-doped Na_5_Lu_9_F_32_/LiYF_4_ single crystals under 980 nm excitation.

Having analyzed all the results above, pump-power dependent of UC external quantum efficiency (EQE) of Na_5_Lu_9_F_32_: 1 mol% Er^3+^/7.9%mol Yb^3+^ under the excitation of NIR ~980 nm light was measured by an integrating sphere and displayed in Fig. 7(a). Following the 980 nm excitation, it should be noted that the EQE increases with the excitation power density, and the optimum EQE was about 6.80% under 5.5 Wcm^−2^ 980 nm light excitation. And once the power density overpasses 5.5 Wcm^−2^, the EQE of UCL hardly changed. In order to verify this point further, we calculated the theoretical EQE based on steady-state rate equations. On account of the simplified model shown in Fig. 7(a) above, the UC external quantum efficiency (ɳ _IQE_) can be estimated by following equations:

2 $$\begin{array}{c}{c}_{0}{N}_{0}{N}_{Yb1}-{c}_{1}{N}_{1}{N}_{Yb2}-{W}_{1}{N}_{1}=0\\ {c}_{1}{N}_{1}{N}_{Yb2}-{W}_{2}{N}_{2}=0\\ {N}_{0}+{N}_{1}+{N}_{2}={N}_{er}\\ {N}_{Yb1}+{N}_{Yb2}={N}_{Yb}\\ {N}_{Yb2}={\rho }_{P}\sigma {N}_{Yb1}\end{array}$$

3 $${\rho }_{P}=\frac{{\lambda }_{P}{I}_{P}}{hc\pi {\omega }_{p}\wedge 2}=\frac{{\lambda }_{p}}{hc}P$$

4 $${N}_{2}=\frac{{c}_{o}{c}_{1}{N}_{c}{N}_{Yb}\wedge 2{\rho }_{P}\wedge 2\sigma \wedge 2}{[{W}_{1}{W}_{2}+{c}_{0}{c}_{1}{N}_{Yb}\wedge 2+({c}_{0}+{c}_{1}){N}_{Yb}]\rho \wedge 2\sigma \wedge 2+[({c}_{0}+{c}_{1}){N}_{Yb}{W}_{2}+2{W}_{1}{W}_{2}]\rho \sigma +{W}_{1}{W}_{2}}$$

5 $$\eta =\frac{{W}_{2}{N}_{2}}{{\rho }_{P}\sigma {N}_{Yb0}}=\frac{4.38P}{1+0.51P} \% $$

where c_0_ and c_1_ are the ET coefficients for the UC processes between the donor and the acceptor in ^4^I_15/2_ and ^4^I_9/2_ states, respectively. N_2_, N_1_, and N_0_ are the population ^2^H_11/2_/^4^S_3/2_^4^,I_9/2_, and ^4^I_15/2_ densities of the levels of the Er^3+^ ions. And the N_Yb1_ and N_Yb2_ represent the ^2^F_7/2_ and ^2^F_5/2_ levels of Yb^3+^ ions, respectively. N_Yb_ and Ner are the concentrations of the Yb^3+^ ions and the Er^3+^ ions. W_1_ denotes the non-radiative relaxation rate from level ^4^I_11/2_ to lower state of Er^3+^, W_2_ is the radiative decay rate from level ^2^H_11/2_/^4^S_3/2_ to ground state of Er^3+^, and σ is the Yb^3+^ absorption cross section at the pumping wavelength. The symbol ρ_P_ denotes the excitation power variable, given by equation (3). Here, I_P_ is the incident pump power, λ_P_ and ω_P_ are the pump wavelength and beam radius, respectively, *h* is Planck’s constant, and c is the speed of light. P is the incident pump power density. From the above equations, we can obtain the population of ^2^H_11/2_/^4^S_3/2_ level using equation (4). Thus, the UC external quantum efficiency (EQE) can be deduced by equation (5). The calculated results were also plotted a line in Fig. 7(a). It can be noted that the theoretical measurement in accordance with the experimental dates. Generally, there exists the thermal effect in the nano-crystals^21, 22^, which results into reduction of the quantum efficiency. It can be confirmed that the rare earth ion doped Na_5_Lu_9_F_32_ single crystal has advantage of achieving high quantum efficiency and high thermal stability compared with its nano-crystal. The inset of the Fig. 7(a) shows the integrated external quantum efficiency on Yb^3+^ concentrations. The relative amount of Yb^3+^ ions tends to improve the EQE in Er^3+^/Yb^3+^ co-doped Na_5_Lu_9_F_32_ single crystals obviously, thus we have investigated the impact of various ytterbium concentrations on the EQE of the up-conversion progress under the same experiment condition. It is shown in the inset of Fig. 7(a) that EQE of up-conversion increases as the Yb^3+^ concentration increases monofonically and reaches its maximum value at ~6.80% when Yb^3+^ concentration is 7.97 mol%. To testify the good UCL effect in Er^3+^/Yb^3+^ co-doped Na_5_Lu_9_F_32_ single crystals, we also compare them with Er^3+^/Yb^3+^ co-doped LiYF_4_ samples in Fig. 7(b) whose maximum EQE of up-conversion is about ~5.8%^23^. It is known that β- NaYF_4_ is widely accepted as the most efficient up-conversion host in the world. The Er^3+^/Yb^3+^(2 mol%/18 mol%) doped β- NaYF_4_ nano-crystal reaches its maximum QY at ~4.8%^24^. It should be noticed that Er^3+^/Yb^3+^ co-doped Na_5_Lu_9_F_32_ single crystals can achieve higher external quantum efficiency at the lower power density, which indicates the Er^3+^/Yb^3+^ co-doped Na_5_Lu_9_F_32_ single crystals own a better UCL effect and worth of further study.

### CIE chromaticity coordinates of Er^3+^/Yb^3+^ co-doped Na_5_Lu_9_F_32_ single crystal samples

**Figure 8:**
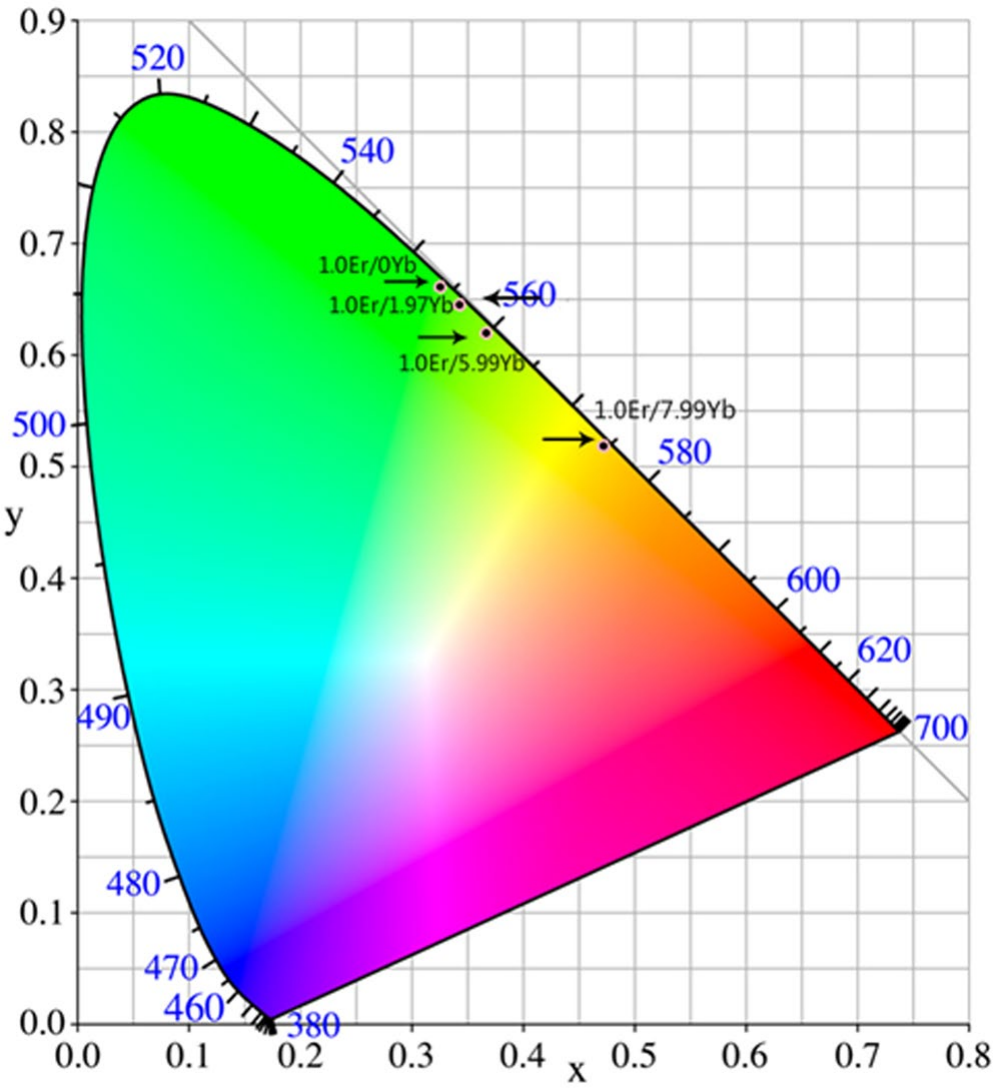
CIE chromaticity coordinates of Er^3+^/Yb^3+^ co-doped Na_5_Lu_9_F_32_ single crystals.

Since the observed UC emissions are located in visible wavelength area, the exact UC luminescence CIE chromaticity coordinates of all the Er^3+^/Yb^3+^ co-doped Na_5_Lu_9_F_32_ single crystal samples are shown in Fig. 8 to obtain the true color of the UC emissions. The CIE coordinates (*x*, y) are (0.3254, 0.6612), (0.3422, 0.6450), (0.3666, 0.6202), and (0.4721, 0.5188) respectively, for Er^3+^/Yb^3+^ co-doped samples with 1.0Er/0Yb, 1.0Er/1.97Yb, 1.0Er/5.99Yb, and 1.0Er/7.97Yb doping concentrations. From the CIE chromaticity coordinates, it is clear that the combination of the UC emission is yellowish green color to which human eyes are sensitive. This characteristic is favorable for applications in UC displays for electro-optical devices.

## Conclusions

Following excitation with a 980 nm diode laser, the Er^3+^/Yb^3+^ co-doped Na_5_Lu_9_F_32_ single crystals can be grown by a vertical Bridgman method and an enhanced up-conversion green and red lights can be obtained. Study on the pump power dependent UC spectra shows that the UC emissions of the green and red lights arise from two-photon process from excited Yb^3+^ to Er^3+^ energy transfer. The combination of the UC green and red lights yields a yellowish green light to which human eyes are very sensitive. The up-conversion quantum efficiency of Er^3+^/Yb^3+^ co-doped Na_5_Lu_9_F_32_ single crystal under 5.5 Wcm^−2^ 980 nm light excitation was as high as 6.80%. Such Er^3+^/Yb^3+^ co-doped Na_5_Lu_9_F_32_ single crystals may have potential applications in the fields of biosensors, infrared pumped visible eye-safe lasers, optical telecommunication and UC displaying devices due to its low phonon energy, high luminous efficiency, high transmittance for light and stability in chemical-physical properties.

## Experimental

### Materials

Er^3+^/Yb^3+^ co-doped samples were grown by an improved Bridgman method according to the formula 30NaF-18KF-(52-χ-γ)YF_3_-χErF_3_-γYbF_3_ (χ = 1.0, γ = 0, 2 6, 8), respectively, with 99.99% purity raw materials of NaF, LuF_3_, ErF_3_, and YbF_3_.

### Synthesis of Na_5_Lu_9_F_32_: Er^3+^/Yb^3+^ single crystals

Then these mixture samples were ground thoroughly in a mortar for about 0.5 h. In order to remove the moisture and the oxide in the raw materials, the mixtures were sintered with anhydrous HF at 750 °C for 8 h. The seed crystals were oriented along a-axis. The seeding temperature was about 770–820 °C and the temperature gradient cross solid-liquid interface was 70–90 °C/cm. The growing process was carried out by lowering the crucible at a rate of 0.05–0.06 mm/h. The detailed process was similar to that reported elsewhere^25^. Figure 1(a) shows the boule of as-grown single crystal and the polished slice of crystal with 1.50mm thickness. The single crystal appears high transparency and pink.

### Characterization

X-ray diffraction (XRD) of the samples was measured using a D8 Advance diffractometer (BRUKER, German). The absorption spectra were recorded with a Cary 5000 UV/VIS/NIR spectrophotometer (Agilent Co., America). The emission spectra were obtained with a FLSP 920 type spectrometer (Edinburgh Co., England). The external quantum efficiency were measured by a fluorescence spectrometer (FLS 980) of Edinburgh instruments combined with an integrating sphere. All the measurements were measured at room temperature. The actual concentrations of Er^3+^ and Yb^3+^ ions in all the samples were measured by an inductively coupled plasma atomic emission spectroscopy (ICP-AES, Perk in Elmer Inc., Optima 3000). The measured Er^3+^ and Yb^3+^ concentrations in all crystals are presented in Table 1.

**Table 1:** The measured molar concentrations and the number of Er^3+^ and Yb^3+^ in Na_5_Lu_9_F_32_ crystal.

Samples	Er^3+^		Yb^3+^	
	Molar concentration (mol%)	The number of ion(10^20^ ions/cm^−3^)	Molar concentration (mol%)	The number of ion (10^20^ ions/cm^−3^)
NFEY1	0.98	1.512	0	0
NFEY2	0.99	1.503	1.97	1.978
NFEY6	1.01	1.502	5.99	6.028
NFEY8	0.99	1.506	7.97	8.02
